# Combined brain and thoracic trauma surgery in a hybrid emergency room system: a case report

**DOI:** 10.1186/s12893-021-01218-y

**Published:** 2021-04-27

**Authors:** Daiki Wada, Koichi Hayakawa, Fukuki Saito, Kazuhisa Yoshiya, Yasushi Nakamori, Yasuyuki Kuwagata

**Affiliations:** 1 Department of Emergency and Critical Care Medicine, Kansai Medical University General Medical Center, 10-15 Fumizono-cho, Moriguchi, Osaka 570-8507 Japan; 2 Coordination Office for Emergency Medicine and International Response, Nagasaki University, 1-7-1 Sakamoto, Nagasaki, Nagasaki 852-8501 Japan; 3 Department of Emergency and Critical Care Medicine, Kansai Medical University Hospital, 2-3-1 Shinmachi, Hirakata, Osaka 573-1191 Japan

**Keywords:** Hybrid emergency room (Hybrid ER), Thoracic endovascular aneurysm repair (TEVAR), Intracranial pressure (ICP), Cerebral perfusion pressure, Traumatic brain injury

## Abstract

**Background:**

A novel trauma workflow system called the hybrid emergency room (Hybrid ER), which combines a sliding CT scanner system with interventional radiology features (IVR-CT), was initially instituted in our emergency department in 2011. Use of the Hybrid ER enables CT diagnosis and emergency therapeutic interventions without transferring the patient to another room. We describe an illustrative case of severe multiple blunt trauma that included injuries to the brain and torso to highlight the ability to perform multiple procedures in the Hybrid ER.

**Case presentation:**

A 46-year-old man sustained multiple injuries after falling from height. An early CT scan performed in the Hybrid ER revealed grade IIIa thoracic aortic injury, left lung contusion, and right subdural haematoma and subarachnoid haemorrhage. Without relocating the patient, all definitive procedures, including trepanation, total pneumonectomy, and thoracic endovascular aneurysm repair were performed in the Hybrid ER. At 5.72 h after definitive surgery was begun, the patient was transferred to the intensive care unit.

**Conclusions:**

The Hybrid ER has the potential to facilitate the performance of multiple definitive procedures in combination to treat severe multiple blunt trauma including injuries to the brain and torso. Emergency departments with more than one resuscitation room would benefit from a Hybrid ER to treat complex emergency cases.

## Background

**Fig. 1:**
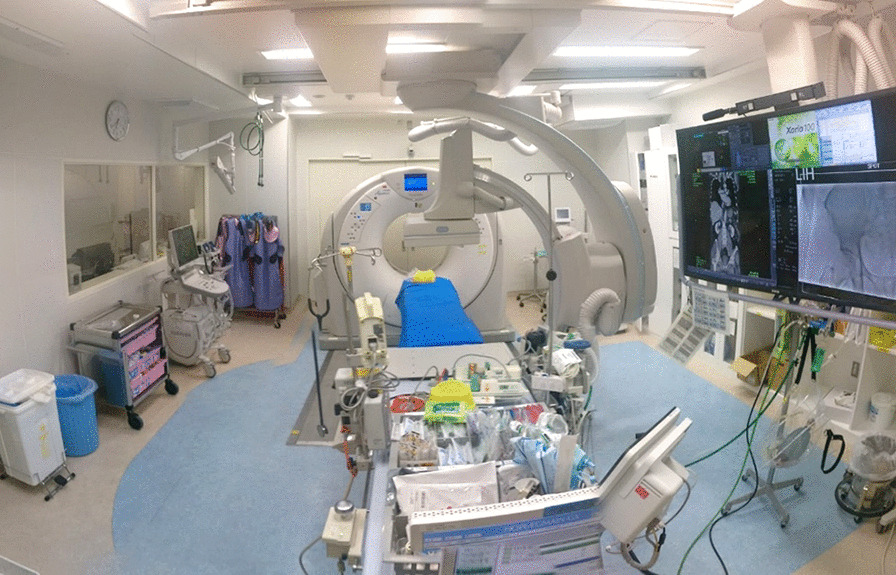
Photograph showing our IVR-CT system in the Hybrid ER (Aquilion PRIME, TSX-303B; Toshiba Medical Systems Corp., Tochigi, Japan). All life-saving procedures including airway management, emergency surgery, and transarterial embolization can be performed on the table without relocating the patient. **a** Sliding CT scanner with the two rows of rails on the floor, **b** CT examination and intervention table, **c** moveable C-arm, **d** 56-inch monitor screen, **e** ultrasonography equipment, and **f** mechanical ventilator. Unlike the usual CT scanner, after the patient is positioned on the CT examination and intervention table, the CT scanner moves to the table on rails with the table fixed. We use the room next to the Hybrid ER for surgical instrument storage and bring only what we need for the surgery into the Hybrid ER. The ventilator is located on the foot side of the trauma table. The blood refrigerator is located in the intensive care unit near the Hybrid ER. When an anaesthesiologist performs inhalation anaesthesia, the anaesthesia machine is located near the head side of the trauma table. Because the sliding CT scanner is moved to the adjacent CT suite after the CT scan, adequate space is available near the head side of the trauma table

A novel trauma workflow system comprising a sliding CT scanner system with interventional radiology features (IVR-CT), which we call the Hybrid emergency room (Hybrid ER), was installed in our hospital’s emergency department (Fig. 1) [1]. Use of the Hybrid ER enables emergency procedures, including damage control surgery and endovascular intervention after CT diagnosis, to be performed without transferring the patient to the CT suite, radiology department, or the operating room. It was previously reported in a retrospective cohort study that trauma workflow in the Hybrid ER may improve survival in patients with severe trauma [2]. Presently, 11 trauma centres have installed a Hybrid ER in Japan [3]. Hybrid ER use reportedly has also allowed safe and prompt initiation of other procedures such as extracorporeal membrane oxygenation [4].

Preventable trauma deaths are defined as those in which significant delays occur before exsanguinating haemorrhage is controlled [5]. Therapeutic procedures and diagnostic evaluation must be concomitantly performed by a multidisciplinary team [6]. Time is crucial in the successful early management of the multiple trauma patient [7]. The Hybrid ER has the potential to resolve the time delay to CT scanning and definitive therapy. Here, this is an illustrative case of severe multiple blunt trauma treated by a combination of total pneumonectomy, thoracic endovascular aneurysm repair (TEVAR), and trepanation for subdural haematoma (SDH) along with intracranial pressure (ICP) monitoring in the Hybrid ER.

## Case presentation

**Fig. 2:**
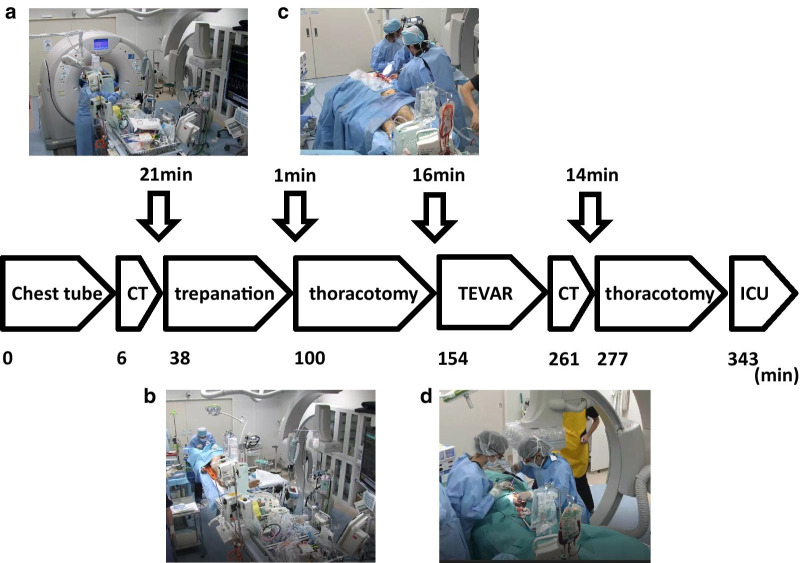
Timeline indicating the time of initiation of CT scanning and emergency procedures, including damage control surgery and endovascular intervention, in the Hybrid ER. The black arrow indicates the time required for preparation until initiation of the next procedure. Photograph **a** showing early CT examination performed by the sliding CT scanner on the same trauma table. Photograph **b** showing trepanation and ICP sensor placement for SDH and SAH performed by one brain surgeon. Photograph **c** showing thoracotomy for left lobe resection performed by three chest surgeons. Photograph **d** showing TEVAR for thoracic aorta injury performed by four vascular surgeons. These procedures were performed sequentially at different times. During surgeries, at least two emergency physicians always perform anaesthesia management. ER and trauma physicians can also perform cardiorespiratory management and administer anaesthesia while the others are engaged in surgery. The sliding CT scanner is moved to the adjacent CT suite during operative procedures

**Fig. 3:**
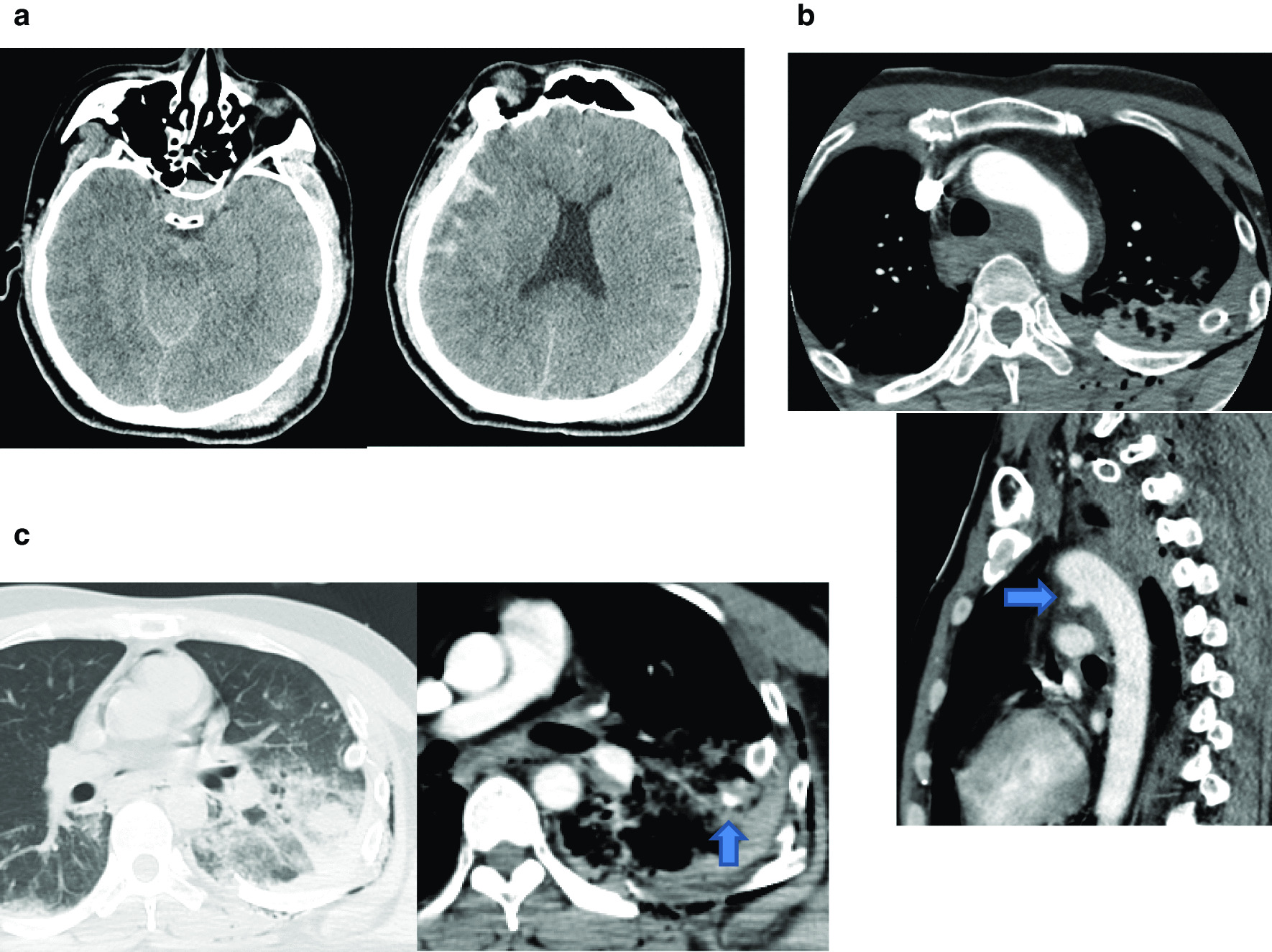
Cranial and thoracic CT images. **a** Right SDH and SAH with brain swelling. **b** Thoracic aorta injury with pseudoaneurysm indicated by the blue arrow. **c** Left lung contusion with contrast agent leakage indicated by the blue arrow

A 46-year-old man sustained multiple injuries after falling from height while working. He was previously healthy and without comorbidities. An emergency physician intubated him on the scene due to consciousness disorder. The patient’s vital signs on arrival were Glasgow Coma Scale, 3; blood pressure, 93/85 mmHg; pulse rate, 156/min; respiratory rate, 22/min; and pulse oximetry of 99% at a FIO_2_ of 1.0 while intubated. Physical examination revealed subcutaneous emphysema over the left chest. A chest tube was inserted for left tension pneumothorax, and a large amount of air and bloody pleural effusion was drained. Thereafter, his blood pressure improved to 102/87 mmHg, but his pulse rate was still over 150/min. Although the patient was not hemodynamically stable, the CT scanner in the Hybrid ER was available. 6 min after admission, a CT examination was begun using the sliding CT scanner with the patient on the same trauma table without relocating him (Fig. 2a). CT revealed a grade IIIa thoracic aortic injury with pseudoaneurysm, left lung contusion with contrast agent leakage, left multiple rib fractures and haemopneumothorax, and right SDH and subarachnoid haemorrhage (SAH) with brain swelling (Fig. 3). Generally, when treating a severely traumatised patient, torso haemostasis is given priority over the treatment of brain injury. However, the CT findings in this patient indicated that treatment for brain injury had the highest priority. At 38 min after admission, the SDH was punctured and an ICP of approximately 10 mmHg was measured (Fig. 2b). Following this surgery, the amount of air and bloody pleural effusion had clearly increased. In addition to CT findings of contrast agent leakage in the left lung contusion, and as bloody sputum was suctioned from the tracheal tube, we determined that a left lung injury was likely the primary source of bleeding. Thus, one minute after trepanation for SDH was completed, left thoracotomy was performed, again without relocating the patient (Fig. 2c). The upper left lobe of the lung was resected as this was the main source of bleeding. However, bleeding from the hilum persisted, which led to the performance of TEVAR for a probable aortic injury (Fig. 2d). Sixteen minutes after the thoracotomy was completed, TEVAR was initiated, with access for the delivery device obtained via the right femoral artery. A grade IIIa thoracic pseudoaneurysm of 13 mm in diameter was located at the isthmus of the aorta. As the left subclavian artery was near the isthmus injury, the stent graft was deployed to extend between the aortic arch and descending aorta, which covered the left subclavian artery, to obtain an adequate proximal landing zone. The proximal end of the stent graft was positioned 6 mm from the left carotid artery, which was preserved. Following these procedures, which the patient tolerated well, another CT scan was performed without relocating the patient and showed no signs of worsening brain injury. Because the amount of bloody pleural effusion continued to increase, repeat left thoracotomy revealed bleeding from the left lower lobe, which was subsequently resected. Thirty units each of whole blood and fresh frozen plasma and 10 units of platelets were transfused. At 5.72 h after initiation of the definitive surgeries and completion of all procedures in the Hybrid ER, the patient was transferred to the intensive care unit.

Decompressive craniectomy on postoperative day 2 and decompressive brain resection on postoperative day 3 were performed for refractory intracranial hypertension. The patient’s Injury Severity Score was 54, Revised Trauma Score was 4.09, and the probability of survival by the Trauma and Injury Severity Score method was 16.

A tracheotomy was performed on postoperative day 13, and he was weaned off mechanical ventilation on postoperative day 14. After undergoing in-patient rehabilitation, he was transferred to another hospital for rehabilitation in postoperative month 5 with a Glasgow outcome scale of 4. At the transfer to the nursing facility, he no longer required mechanical ventilation.

## Discussion and conclusions

Whole-body computed tomography (WBCT) is important in the early diagnostic phase of trauma care, and its benefits for patient survival have been suggested by several studies [8, 9]. CT performed before emergency bleeding control might reduce mortality, especially in haemodynamically unstable patients and severe trauma patients with a Trauma and Injury Severity Score probability of survival of < 50% [10]. Several studies reported that CT installed in the ER has become important for early diagnosis in trauma care [11–13]. Although this concept improves the disadvantage of delayed CT diagnosis, patient transfer to the operating room or the angiography room remain as problems requiring resolution. Therefore, we implemented a novel concept called the Hybrid ER that comprises a sliding CT scanner system with IVR features whose use enables early CT diagnosis and emergency therapeutic intervention without relocating the patient (Fig. 1).

Severe multiple trauma is often associated with lung injury and presents with a wide spectrum of severity [14]. Blunt thoracic trauma and structural damage to vital organs within the thoracic region substantially influence morbidity and mortality in multiple trauma patients [15]. Among deaths in severely injured patients, 20 to 25% are attributed to chest injury [16, 17]. In addition, pulmonary contusion is present in 29% of patients with traumatic brain injury (TBI) [18]. Chest trauma resulting in hypoxemia, constituting a secondary insult, may be the leading cause that aggravates TBI [19].

Control of the present patient’s haemodynamics and cerebral perfusion was potentially difficult because the severe lung contusion could lead to haemorrhagic shock, the blunt thoracic aortic injury would require control of blood pressure to prevent rupture of the pseudoaneurysm, and TBI would require an optimal cerebral perfusion pressure (CPP) for neuroprotective therapy. In severe TBI patients, the main aim of intensive care management is to prevent secondary injury [20], and central to this is the control of ICP and maintenance of an adequate CPP [21]. Thus, the trauma team initiated ICP monitoring and maintenance of the CPP level first, treated the lung injury to control thoracic haemorrhage second, and performed TEVAR as the third step.

This patient required determination of treatment priority and performance of damage control surgery and endovascular intervention for the thoracic injuries and management of TBI in adequate order without wasting time. Use of the Hybrid ER allowed multiple emergency bleeding controls to be applied in more than one body region of a patient. Kinoshita et al. suggested that concurrent performance of bleeding control procedures and ICP monitoring would be feasible in Hybrid ER settings for multiple trauma patients with exsanguinating haemorrhage and TBI [22]. Because use of the Hybrid ER allowed CT examinations before intervention, except when cardiac arrest was imminent, this trauma workflow was actually realised in our patient. According to the ATLS algorithm, patient resuscitation has priority over advanced diagnostic procedures including CT scanning [23]. When hemodynamically unstable, the patient is examined clinically and undergoes conventional radiography, ultrasonography, and emergency surgery before CT scanning. Even if the patient is not hemodynamically stable, workflow in the Hybrid ER permits the performance of WBCT and determination of the priority of definitive procedures for severe injuries including TBI. However, if CT scanning is not available to determine the reason for hemodynamical instability, we perform emergency surgery and intervention before the CT scan.

To our knowledge, few cases of multiple severe thoracic injuries and TBI require a combination of IVR procedures and invasive surgical interventions in the emergency department. The good outcome in the present patient may have been obtained by safely introducing appropriate therapies to treat his multiple lethal injuries in the Hybrid ER. The disadvantage of the Hybrid ER is that the room might be occupied for a long time when performing multiple definitive procedures on one severely injured patient, and this could affect the efficacy of other in/outpatient workflow. In 2017, we installed a new CT suite with a radiolucent table next to the Hybrid ER [24]. These two rooms are separated by a moveable door, and the sliding CT scanner can be moved between these two rooms depending on need. When emergency surgery or IVR procedures are performed in the Hybrid ER, the sliding CT scanner is moved to the new CT suite, where CT scanning of another in/outpatient can be done. The emergency department has two conventional resuscitation rooms next to the Hybrid ER in which emergency surgery can be performed on other patients. We have not yet treated an adequate number of patients with multiple severe injuries to the brain and torso to clarify the safety of performing multiple treatments in the Hybrid ER. If the sophistication of the trauma teams can be improved, some procedures could be performed simultaneously or overlap during other surgery. Our goal is to establish a well-trained trauma team that can perform multiple procedures simultaneously or overlap them in the Hybrid ER. To further reveal the efficiency of the Hybrid ER system, additional cases in which definitive interventions were performed need to be collected and evaluated. A protocol for patient selection for admittance to the Hybrid ER or conventional resuscitation room that improves the efficacy of other in/outpatient workflow is also required. The Hybrid ER has the potential to facilitate the performance of multiple definitive procedures in combination to treat severe multiple blunt trauma including injuries to the brain and torso.

## Data Availability

This case report only contains clinical data from the medical records of the patient reported herein. The data will be made available upon request.

## Abbreviations

CPP: Cerebral perfusion pressure
CT: Computed tomography
WBCT: Whole-body computed tomography
Hybrid ER: Hybrid emergency room
ICP: Intracranial pressure
IVR: Interventional radiology
SDH: Subdural haematoma
SAH: Subarachnoid haemorrhage
TBI: Traumatic brain injury
TEVAR: Thoracic endovascular aneurysm repair

## References

[1] Wada D, Nakamori Y, Yamakawa K, Fujimi S (2012). First clinical experience with IVR-CT system in the emergency room: positive impact on trauma workflow. Scand J Trauma Resusc Emerg Med.

[2] Kinoshita T, Yamakawa K, Matsuda H, Yoshikawa Y, Wada D, Hamasaki T (2019). The survival benefit of a novel trauma workflow that includes immediate whole-body computed tomography, surgery, and interventional radiology, all in one trauma resuscitation room: a retrospective historical control study. Ann Surg.

[3] Watanabe H, Shimojo Y, Hira E, Kuramoto S, Muronoi T, Oka K (2018). First establishment of a new table-rotated-type hybrid emergency room system. Scand J Trauma Resusc Emerg Med.

[4] Miyazaki K, Hikone M, Kuwahara Y, Ishida T, Sugiyama K, Hamabe Y (2019). Extracorporeal cardiopulmonary resuscitation for massive pulmonary embolism in a "hybrid emergency room". Am J Emerg Med.

[5] Tien HC, Spencer F, Tremblay LN, Rizoli SB, Brenneman FD (2007). Preventable deaths from hemorrhage at a level I Canadian trauma center. J Trauma.

[6] Philipp MO, Kubin K, Hörmann M, Metz VM. Radiological emergency room management with emphasis on multidetector-row CT. Eur J Radiol. 2003; 48: 2-4. 10.1016/s0720-048x(03)00206-7 14511855

[7] Bunya N, Harada K, Kuroda Y, Toyohara T, Toyohara T, Kubota N (2017). The effectiveness of hybrid treatment for sever multiple trauma: a case of multiple trauma for damage control laparotomy and thoracic endovascular repair. Int J Emerg Med.

[8] Huber-Wagner S, Lefering R, Qvick LM, Körner M, Kay MV, Pfeifer KJ (2009). Effect of whole-body CT during trauma resuscitation on survival: a retrospective, multicentre study. Lancet.

[9] Yeguiayan JM, Yap A, Freysz M, Garrigue D, Jacquot C, Martin C (2012). Impact of whole-body computed tomography on mortality and surgical management of severe blunt trauma. Crit Care.

[10] Wada D, Nakamori Y, Yamakawa K, Yoshikawa Y, Kiguchi T, Tasaki O (2013). Impact on survival of whole-body computed tomography before emergency bleeding control in patients with severe blunt trauma. Crit Care.

[11] Hilbert P, Zur Nieden K, Hofmann GO, Hoeller I, Koch R, Stuttmann R (2007). New aspects in the emergency room management of critically injured patients: a multi-slice CT-oriented care algorithm. Injury.

[12] Fung Kon Jin PH, Gostlings JC, Ponsen KJ, van Kuijk C, Hoogerwerf N, Luitse JS (2008). Assessment of a new trauma workflow concept implementing a sliding CT scanner in the trauma room: the effect on workup times. J Trauma..

[13] Wurmb TE, Frühwald P, Hopfner W, Keil T, Kredel M, Brederlau J (2009). Wholebody multislice computed tomography as the first line diagnostic tool in patients with multiple injuries: the focus on time. J Trauma.

[14] Ried M, Bein T, Philipp A, Müller T, Graf B, Schmid C (2013). Extracorporeal lung support in trauma patients with severe chest injury and acute lung failure: a 10-year institutional experience. Crit Care.

[15] Huber S, Biberthaler P, Delhey P, Trentzsch H, Winter H, van Griensven M (2014). Trauma Register DGU Predictors of poor outcomes after significant chest trauma in multiply injured patients: a retrospective analysis from the German Trauma Registry (Trauma Register DGU®). Scand J Trauma Resusc Emerg Med..

[16] Gaillard M, Hervé C, Mandin L, Raynaud P (1990). Mortality prognostic factors in chest injury. J Trauma.

[17] Inthorn D, Huf R (1992). Thoracic trauma in multiple trauma. Anasthesiol Intensivmed Notfallmed Schmerzther.

[18] Hyder AA, Wunderlich CA, Puvanachandra P, Gururaj G, Kobusingye OC (2007). The impact of traumatic brain injuries: a global perspective. NeuroRehabilitation.

[19] Dai D, Yuan Q, Sun Y, Yuan F, Su Z, Ding J (2013). Impact of thoracic injury on traumatic brain injury outcome. PLoS ONE.

[20] Beqiri E, Smielewski P, Robba C, Czosnyka M, Cabeleira MT, Tas J (2019). Feasibility of individualised severe traumatic brain injury management using an automated assessment of optimal cerebral perfusion pressure: the COGiTATE phase II study protocol. BMJ Open.

[21] Stocchetti N, Carbonara M, Citerio G, Ercole A, Skrifvars MB, Smielewski P (2017). Severe traumatic brain injury: targeted management in the intensive care unit. Lancet Neurol.

[22] Kinoshita T, Yamakawa K, Yoshimura J, Watanabe A, Matsumura Y, Ito K (2018). Japanese Association for Hybrid Emergency Room System (JA-HERS) Scientific Promotion Committee. First clinical experiences of concurrent bleeding control and intracranial pressure monitoring using a hybrid emergency room system in patients with multiple injuries. World J Emerg Surg..

[23] Committee on Trauma (2009). American College of Surgeons: Advanced Trauma Life Support (ATLS) for Physicians.

[24] Wada D, Nakamori Y, Kanayama S, Maruyama S, Kawada M, Iwamura H (2018). First installation of a dual-room IVR-CT system in the emergency room. Scand J Trauma Resusc Emerg Med.

